# Gold Nanohole Array with Sub-1 nm Roughness by Annealing for Sensitivity Enhancement of Extraordinary Optical Transmission Biosensor

**DOI:** 10.1186/s11671-015-0944-x

**Published:** 2015-05-27

**Authors:** Jian Zhang, Mehrdad Irannejad, Mustafa Yavuz, Bo Cui

**Affiliations:** Waterloo Institute for Nanotechnology (WIN), University of Waterloo, Waterloo, ON N2L 3G1 Canada; INPAC-Institute for Nanoscale Physics and Chemistry and Physics Department, KU Leuven, Celestijnenlaan 200 D, B-3001 Leuven, Belgium

**Keywords:** Extraordinary optical transmission, Sensitivity, 16-Mercaptohexadecanoic acid, Double liftoff, Annealing, Nanohole array

## Abstract

Nanofabrication technology plays an important role in the performance of surface plasmonic devices such as extraordinary optical transmission (EOT) sensor. In this work, a double liftoff process was developed to fabricate a series of nanohole arrays of a hole diameter between 150 and 235 nm and a period of 500 nm in a 100-nm-thick gold film on a silica substrate. To improve the surface quality of the gold film, thermal annealing was conducted, by which an ultra-smooth gold film with root-mean-square (RMS) roughness of sub-1 nm was achieved, accompanied with a hole diameter shrinkage. The surface sensitivity of the nanohole arrays was measured using a monolayer of 16-mercaptohexadecanoic acid (16-MHA) molecule, and the surface sensitivity was increased by 2.5 to 3 times upon annealing the extraordinary optical transmission (EOT) sensor.

## Background

Plasmonic devices such as plasmonic waveguide [1–3] and bio- or chemical sensors based on localized or propagating surface plasmon resonance [4–7] are based on nanostructured noble metal films including gold, silver, and platinum. Among them, extraordinary optical transmission (EOT) device consisting of periodic nanohole array patterned in noble metal film has been an active research area both theoretically [8–14] and experimentally [15–18] since 1998 when EOT phenomenon was first observed [19]. The EOT phenomenon is attributed to the resonant excitation of a surface plasmon polariton (SPP) and its interference at the two surfaces/interfaces of the hole array [16, 20]. Due to the high sensitivity of the transmission to the refractive index change in the ambient medium [21], one of the most interesting applications of the EOT phenomenon is chemical/bio-sensing [22].

It is challenging to fabricate the nanohole array (NHA) structure by nanolithography and liftoff process because the metal film is too thick for a clean liftoff (typically ≥100 nm for Au and ≥300 nm for Ag to make the film “optically thick”). As a result, focused ion beam (FIB) milling is commonly utilized for NHA fabrication, which is very time-consuming for large-area patterning [23–26], and Ga contamination could be another serious issue [27]. A second method involves the evaporation of noble metal onto a freestanding thin (typically 50–200 nm) Si_3_N_4_ membrane patterned with a through-membrane NHA [28–30]. However, the thin membrane is very brittle and breaks easily.

Besides an efficient fabrication method, the noble metal film quality (surface roughness, grain size, purity, etc.) is equally important for EOT device application. An ideal film should be single crystalline with atomically smooth surface, which maximizes the propagation distance of SPP and thus the transmission spectrum peak amplitude and reduces the linewidth by eliminating light scattering and loss at rough surface and grain boundary [1, 31, 32]. Unfortunately, it is challenging to synthesize or grow uniform single-crystalline film over a large area, and noble metal film is commonly deposited by evaporation or sputtering that, even with an adhesion/seed layer of Ti or Cr, both give a rough or even incontinuous island film.

Various methods have been previously demonstrated to obtain smoother noble metal films. The first method utilized 1-nm germanium [33] and a self-assembled monolayer containing –SH end group [34] as a seed layer, and achieved very smooth (with root-mean-square (RMS) roughness <1 nm) silver and gold film with a thickness of 20 nm. However, for a 100-nm-thick Au film needed for EOT, our results showed insignificant improvement as compared to the film using an adhesion layer of 1-nm chromium or titanium. The second method generated ultra-smooth Au film by mechanical pressing process [35]. For this method, extremely high pressure of 100 ~ 700 MPa is required even for the soft metal Au and Ag (as a comparison, 1 mPa is needed for thermal nanoimprint lithography). Such a high pressure may break the wafer due to trapped dust particles and/or uneven plate surface. The third method is termed as “template stripping”, for which a scotch tape or epoxy is used to peel the film off the substrate, thus exposing the smooth side (in contact with the smooth substrate during film deposition) of the film for device fabrication [36–39]. However, for an EOT device based on transmission measurement, the rough bottom side of the film still affects unfavorably the device performance. The fourth method consists of chemical polishing using mechanical assistance or electrochemical reaction [40, 41], but the film and nanostructure will be attacked during the polishing process. Furthermore, for all the above four methods, only the film surface is considered and there is no improvement to the grain structure of the metal film.

Recently, thermal annealing was applied to improve film smoothness and increase the grain size (thus reduce light scattering and loss at grain boundary) for various plasmonic devices, such as surface plasmon enhanced solar cell [42] and sensors based on metal-enhanced fluorescence emission [43] and localized surface plasmon resonance using nano-disks [44]. In this work, we report the effect of thermal annealing on the performance of EOT sensor, as well as an efficient fabrication approach using electron beam lithography (EBL) followed by a double liftoff process.

## Methods

EBL, which is a very feasible and powerful technique in nanostructure fabrication, was employed for patterning NHA structures on a glass substrate following a double liftoff process. Annealing was applied for improving the surface roughness of the NHAs and increasing the sensitivity of the fabricated structures.

### Double Liftoff Process

**Fig. 1:**
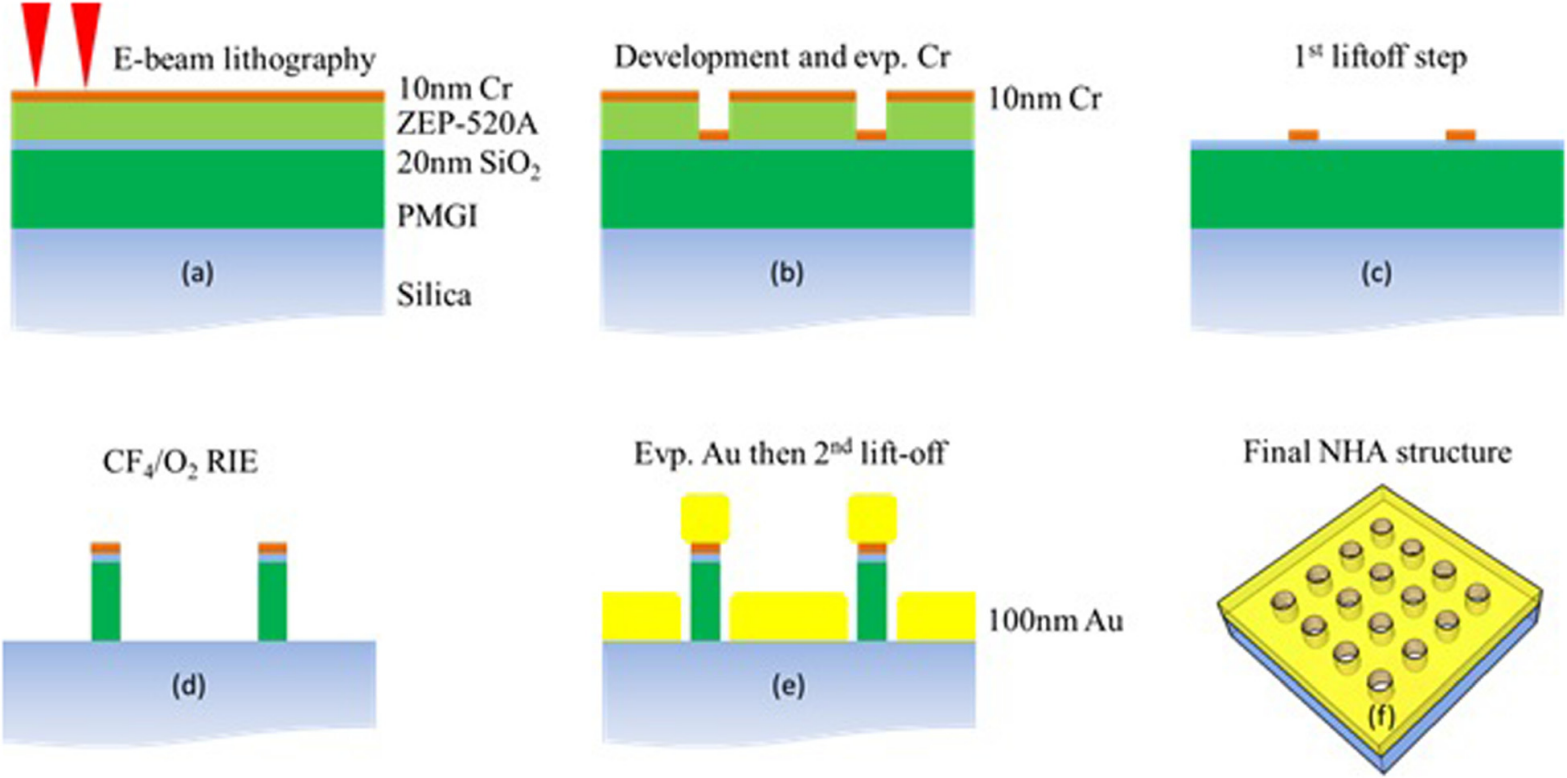
Schematic diagram of NHA fabrication through a double liftoff process. **a** E-beam exposure, **b** development and Cr deposition, **c** first liftoff process, **d** CF_4_ RIE followed by O_2_ RIE, **e** 100-nm gold deposition and second liftoff process, and **f** final NHA structure

NHA structures were fabricated through the double liftoff process as shown in Fig. 1. A cleaned 2-in. silica wafer was sequentially coated with 400-nm poly(dimethyl glutarimide) (PMGI; MicroChem Corp.) by spin coating as the sacrificial layer, 20-nm SiO_2_ by electron beam evaporation as the separating layer, 90-nm ZEP-520A (ZEON Inc.) by spin coating as the resist, and 10-nm Cr by electron beam evaporation as the conductive charge dissipation layer (Fig. 1, step a). EBL was carried out using Raith 150TWO system at an acceleration voltage of 20 kV and a beam current of 0.33 nA. Dot array pattern of a period of 500 nm over an area of 100 μm^2^ was exposed with the typical dose of 40 and 60 fc/dot. The Cr layer was removed by wet etching after EBL, and the patterns were developed using amyl acetate (Sigma-Aldrich) for 1 min and rinsed by fresh isopropanol (Fig. 1, step b). For the first liftoff step, the sample was evaporated by 10-nm Cr and soaked in anisole for 4 h (Fig. 1, step c). Next, by using the Cr as etching mask, the under layers of SiO_2_ and PMGI were etched using reactive ion etching by CF_4_ (20-sccm CF_4_, 20 mTorr, 100-W RF power) and O_2_ (20-sccm O_2_, 20 mTorr, 20-W RF power), respectively (Fig. 1, step d). Finally, 1-nm Cr and 100-nm gold were deposited at a slow rate of 0.2 Å/s to reduce surface roughness using electron beam evaporation, and the sample was soaked in AZ 300 MIF (AZ Electronic Materials USA Corp.) for the second liftoff process (Fig. 1, step e).

The thermal annealing of the fabricated NHAs was carried out using a tube furnace under Ar atmosphere at 600 °C for 1 h to improve the surface morphology of the fabricated structures. The surface morphologies of gold structure before and after annealing were characterized by AFM and SEM.

### Optical Characterization

The optical transmission spectra were recorded using a fiber optical spectrometer (USB4000, Ocean Optics) with a spectral resolution of 1 nm. The fabricated NHAs were illuminated using a tungsten-halogen lamp which provides broadband radiation in the wavelength range of 200 to 900 nm, and a × 10 objective lens of numerical aperture (NA) of 0.15 was used as collimator. The transmitted spectrum was collected using a × 40 objective lens of NA of 0.4 and coupled to the fiber that was connected to the spectrometer. All spectra were recorded by averaging 100 acquisitions each with an integration time of 100 ms.

Both as-fabricated and annealed NHAs were treated by O_2_ plasma and SC-1 (also known as RCA-1) cleaning before each measurement. The 16-MHA (Sigma-Aldrich) was utilized as the target molecule. A drop of 1 mM 16-MHA in ethanol was coated on the NHA surface followed by rinsing with fresh ethanol for three times and drying at room temperature to form a self-assembled monolayer (SAM) of target molecule with the thickness approximately 2.2 nm [45].

### Numerical Simulation

The optical behavior of the incident electromagnetic field through the NHAs was numerically studied using the 3D full wavevector FDTD method, which is a reliable method in solving Maxwell’s equations in dispersive media like gold and silver. Each medium was specified by a relative permittivity, *ε*(*ω*). For glass substrate and superstrate (i.e., target molecule) layers, *ε*(*ω*) were assumed respectively as *n*^2^ (=1.45^2^) and *n*_s_^2^ (=1.48^2^). The Lorentz-Drude model was employed to calculate the permittivity of the film layer [46].

The FDTD was carried out using the commercial software package (FDTD solution 8) from Lumerical Inc. The plane wave source, nanohole structure, and monitor were co-planar with boundary conditions that made them effectively infinite. In this study, a plane wave of wavelength in the range of 300 to 900 nm with an electric field amplitude of 1 V/m that propagates in the *z*-axis was used. Instead of using the ideal white light source coming with the Lumerical FDTD software, we measured the spectrum of our white light source and used the measured spectrum for the simulation, which resulted in a better agreement between the simulation and experimental results. The rough surface was simulated by changing different roughness amplitude of the objects in FDTD solution package from Lumerical. The asymmetric and symmetric boundary conditions were considered for the *x*- and *y*-directions, respectively, and perfect matching layer (PML) was used along the *z*-direction as absorbing boundary condition to study the transmission properties at normal incident of electromagnetic wave through the subwavelength hole structure. The asymmetric and symmetric boundary conditions were chosen in order to reduce the calculation time [47]. The calculation grid resolution was as high as 5 nm (grid point-to-point distance) in the simulation cell, and the conformal mesh method was used to calculate the electric and magnetic field at the corner and rounded region of the nanoholes. The calculation time was set to 150 fs, and the transmission spectra were calculated using an X-Y monitor at 200 nm away from the film/air interface.

## Results and Discussion

### Comparison of Single and Double Liftoff Processes for NHA Fabrication

**Fig. 2:**
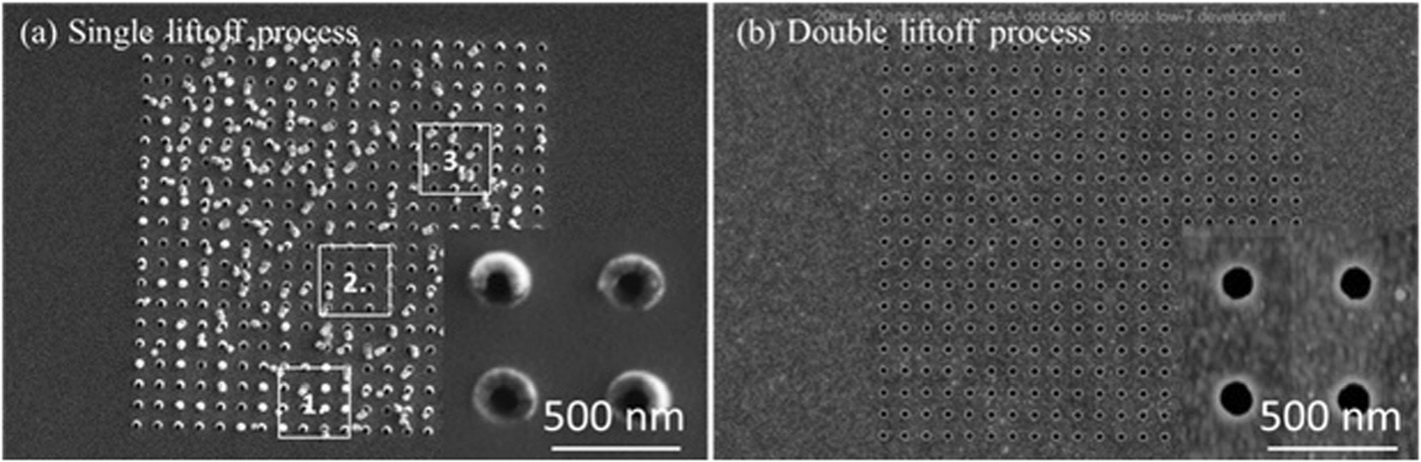
The SEM images of fabricated NHAs of hole diameter of 150 nm and period of 500 nm through **a** conventional single liftoff process and **b** double liftoff process. Three different types of defects were found in conventional liftoff process as marked as region *1*) resist pillars covered by lateral deposition of Au, *2*) nanoholes with chimney shape, and *3*) collapsed pillar. The inset images show the higher magnification of **a** chimney-shape nanoholes and **b** nanohole array of period of 500 nm and hole diameter of 150 nm

The NHA can be fabricated either using a simple liftoff process with negative resist or double liftoff process with positive resist (see the “Methods” section). MaN-2403 (Microresist Technology) was used for the single liftoff process. The SEM images of fabricated NHAs on a 100-nm gold film through single and double liftoff processes were compared in Fig. 2. As can be seen from Fig. 2a, three different types of defects were formed in a single liftoff process. In a single liftoff process, because of the lack of under-cut profile and the very thick metal coating, the sidewall of resist pillars was covered by a thin layer of metal film due to lateral deposition, which prevents the resist dissolution during the liftoff process (region 1 in Fig. 2a). The chimney-shape structures were also observed in the fabricated NHAs utilizing a single liftoff process which was again attributed to the lateral deposition of metallic film on the sidewall of the pillars (region 2 in Fig. 2a). The last defect that was observed in the fabricated NHAs through a single liftoff process was the collapse of the resist pillars (region 3 in Fig. 2a) which was due to the capillary force (during rinse solution drying after development) and high aspect ratio of resist structure. It was found that on using the double liftoff process instead of the conventional one, the aforementioned defects were eliminated and the fabricated NHAs were defect-free as it is shown in Fig. 2b. The inset SEM image of Fig. 2b shows higher magnification of the fabricated NHAs through the double liftoff process.

### Effects of NHA Annealing on Optical Transmission

**Fig. 3:**
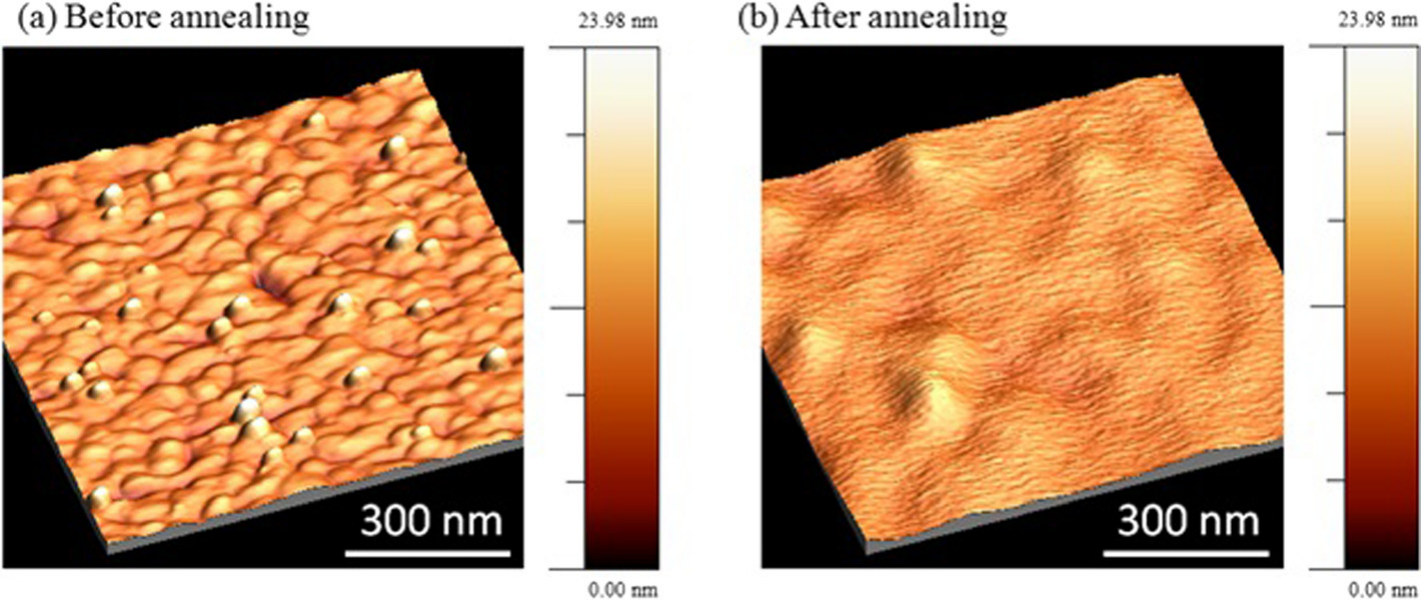
AFM images of the deposited gold film **a** before and **b** after annealing at 600 °C for 1 h. The RMS surface roughness value was reduced from 1.89 to 0.87 nm as measured by AFM

**Fig. 4:**
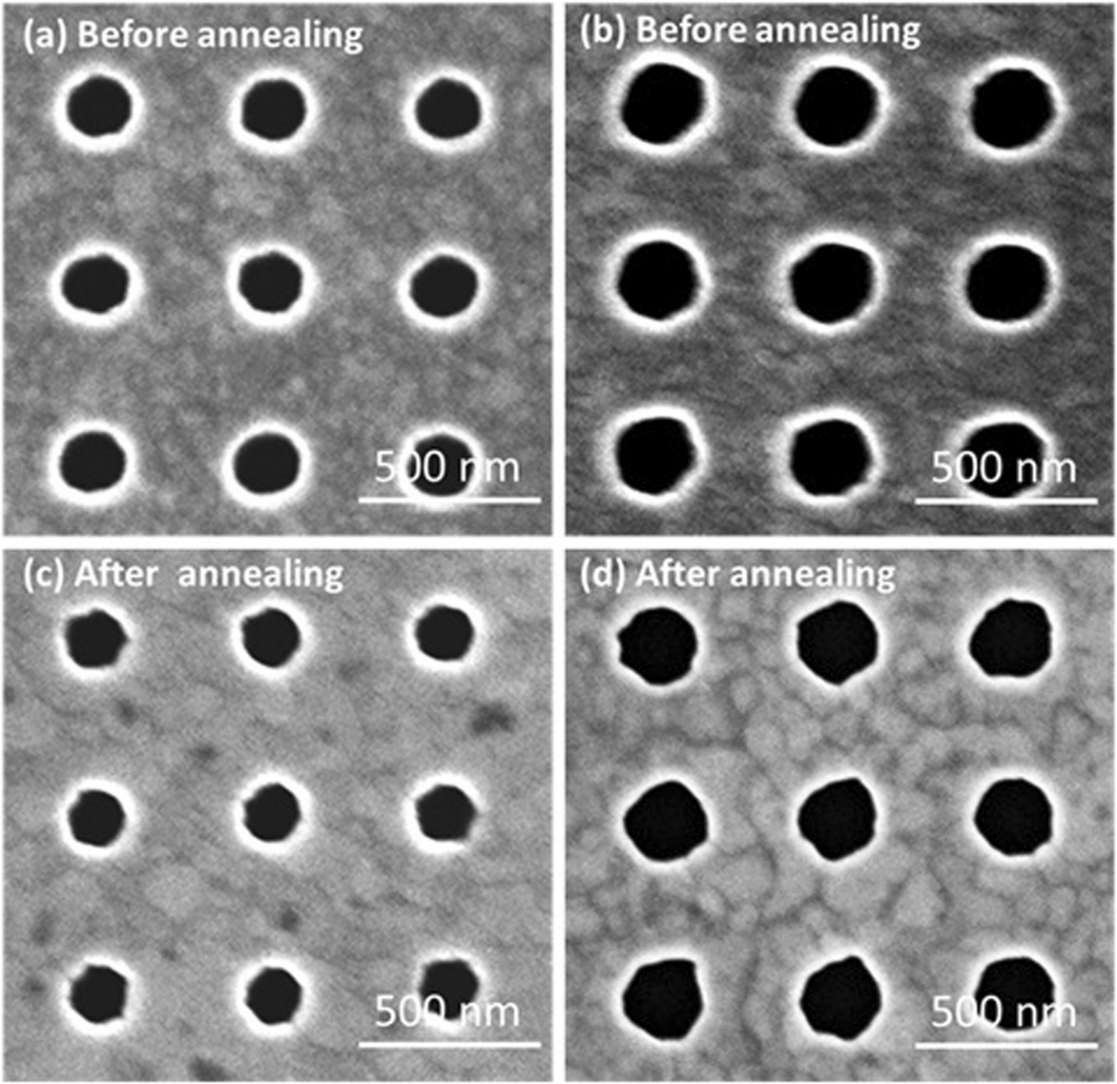
The SEM images of the NHAs (**a**), **b** before and **c**, **d** after annealing with the exposure dose of 40 and 60 fc/dot, respectively. The SEM images show the hole diameter shrank from **a** 175 nm to **c** 150 nm (by 13 %) and from **b** 235 nm to **d** 209 nm (by 11 %), respectively

It was found that an annealing process modified the surface morphology of gold films and nanohole diameter. As it is clear from Fig. 3, the RMS roughness value of the gold film was reduced from 1.89 to 0.87 nm after annealing. The average peak-to-valley height deducted from AFM measurement was reduced from approximately 10 to 2.5 nm. The SEM images of NHAs before and after annealing are shown in Fig. 4, in which the hole diameters after the annealing procedure for the two NHAs were shrunk by 13 and 11 %. It was also found that after annealing the small “islands” on the film surface disappeared and grain boundaries were supposedly increased to produce the more uniform films compared to the non-annealed sample. The increased grain size after annealing was confirmed previously by various methods, including X-ray diffraction [48], scanning tunneling microscopy [49], AFM mapping [50], depth-dependent spectroscopy [51], and FIB imaging [52].

**Fig. 5:**
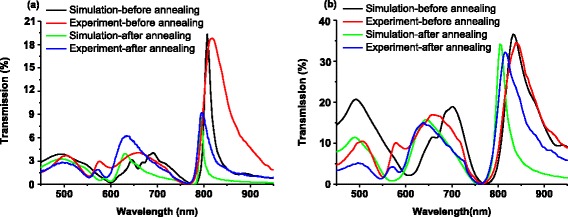
Comparison of optical transmission spectrum of NHAs with the diameters of **a** 175 nm before annealing by simulation (*black line symbol*) and measurement (*red line symbol*), and (same sample shrunk to) 150 nm after annealing by simulation (*green line symbol*) and measurement (blue line symbol); **b** 235 nm before annealing by simulation (*black line symbol*) and measurement (*red line symbol*), and (same sample shrunk to) 209 nm after annealing by simulation (*green line symbol*) and measurement (*blue line symbol*)

To understand the effect of annealing on the optical behavior of fabricated NHAs, the optical transmission of the NHAs with the same period of 500 nm and the hole diameters of 175 nm (before annealing) and 150 nm (same NHA after annealing), and 235 nm (before annealing) and 209 nm (same NHA after annealing) are compared in Fig. 5a, b, respectively. It was found that the (1,0) resonance mode of the annealed NHAs shows a blue shift of 19 and 23 nm which can be attributed to the hole diameter shrinkage from 175 to 150 nm and 235 to 209 nm, respectively. This is because smaller holes lead to higher resonance frequency (blue shift) for the localized surface plasmon, which couples with the propagating surface plasma to determine the overall transmission spectrum [21].

**Fig. 6:**
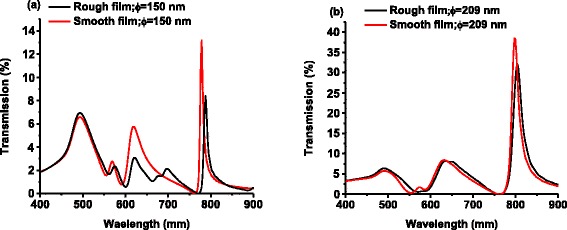
Comparison of simulated optical transmission spectrum of NHAs with diameters *ϕ* of **a** 150 nm without any roughness (mimicking the annealed sample) (*red line symbol*), and with roughness of RMS = 1.89 nm (*black line symbol*); and **b** 209 nm without any roughness (*red line symbol*), and with roughness of RMS = 1.89 nm (*black line symbol*)

To understand the impact of annealing on the quality of fabricated Au NHAs, the transmission spectra of the annealed and non-annealed NHAs with same diameters of 150 and 209 nm are compared by finite-difference time-domain (FDTD) simulation in Fig. 6. Here, the small RMS roughness for the annealed sample is ignored, whereas that for the non-annealed sample is taken as 1.89 nm as measured by AFM. Clearly, the intensity of the annealed NHAs is significantly higher than the non-annealed ones with the same diameter, which can be attributed to the reduced light scattering at the surface for the annealed sample.

### Surface Plasmon Resonance Sensing

**Fig. 7:**
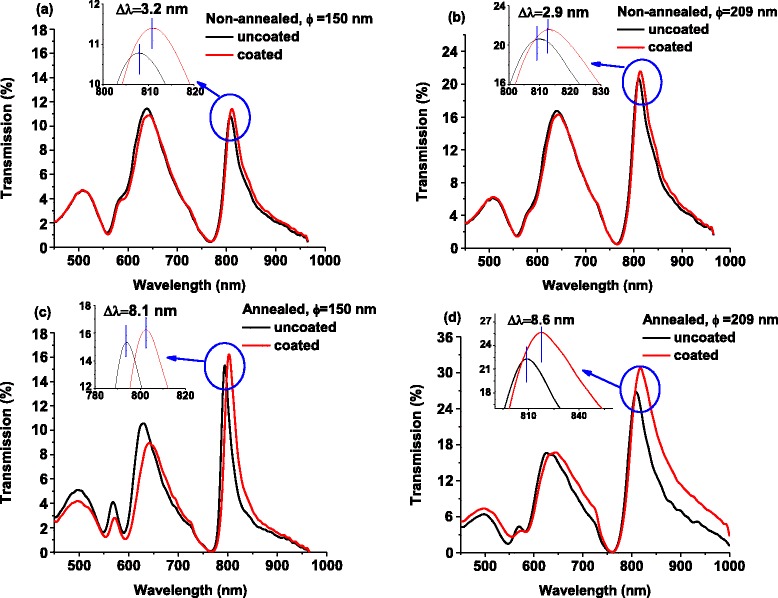
Measured transmission spectrum of coated and uncoated NHAs by a target molecule 16-MHA. **a**, **b** As-fabricated NHAs of hole diameter of 150 and 209 nm, respectively, and **c**, **d** annealed NHAs of hole diameter of 150 and 209 nm, respectively

The effect of annealing on the surface sensitivity of the fabricated NHAs on the silica glass was studied by using ~2.2-nm-thick SAM layer of 16-mercaptohexadecanoic acid (16-MHA) which is one of the most popular target molecules [53–55]. Both of the as-fabricated and annealed NHA structures were coated by 16-MHA, and the recorded optical spectra are compared in Fig. 7. Using the 16-MHA molecule (*n* = 1.48) as sensing medium in the non-annealed NHA, the (1,0) resonance mode surface sensitivity (∆*λ*/∆*n*, ∆*n* = 0.48) was measured as 6.7 and 6.0 nm/refractive index unit (RIU) for the diameter of 150 and 209 nm, respectively. However, for the annealed NHA, the sensitivity was enhanced to 17.9 and 16.9 nm/RIU for the diameter of 209 and 150 nm, respectively. Note that here we have a very low sensitivity because it is *surface* sensitivity (only one monolayer coverage), not *bulk* refractive index sensitivity [56–58]. Both simulated and experimental linewidth (full width at half maximum (FWHM)), surface sensitivity, and figure of merit (FOM; (Δ*λ*/Δ*n*)/FWHM) are compared in Table 1. The 2.5 to 3 times higher sensitivity for annealed NHAs was achieved which could be attributed to the smoother surface area and larger grain size that reduces the surface wave scattering and propagation loss compared to the as-fabricated one [37, 38].

**Table 1:** Simulation and experimental FWHM, surface (monolayer target molecule coverage) sensitivity, and FOM of the NHA sensor before and after annealing. The annealed and as-fabricated NHAs having the same hole diameters of 209 and 150 nm are selected for comparison

Hole diameter	Simulation	Experiment (Δ*n* = 0.48)		
	FWHM (nm)	FWHM (nm)	Surface sensitivity (nm/RIU)	FOM (/RIU)
Before annealing				
209 nm	24.6	77.4	6.0	0.08
150 nm	55.4	64.2	6.7	0.1
After annealing				
209 nm	37.9	58.2	16.9	0.29
150 nm	9.7	31.5	17.9	0.57

## Conclusions

The double liftoff process was developed for nanohole array fabrication in a thick layer (100 nm) of gold for EOT sensing. This technique is versatile for fabrication of many kinds of high-aspect-ratio noble metal structures. The gold film quality of the fabricated NHAs was improved by annealing at 600 °C under Ar atmosphere for 1 h. It was found that the RMS surface roughness value of the deposited gold film was reduced by 54 % and the surface sensitivity was enhanced by 2.5 ~ 3× as result of annealing. The optical transmission spectra of the annealed NHAs were in better agreement with numerical simulated spectra than the non-annealed NHA.
